# The interplay of common genetic variants NRG1 rs2439302 and RET rs2435357 increases the risk of developing Hirschsprung’s disease

**DOI:** 10.3389/fcell.2023.1184799

**Published:** 2023-07-07

**Authors:** Shuiqing Chi, Shuai Li, Guoqing Cao, Jialing Guo, Yunqiao Han, Yun Zhou, Xi Zhang, Yibo Li, Zhibin Luo, Xiangyang Li, Liying Rong, Mengxin Zhang, Linglu Li, Shaotao Tang

**Affiliations:** ^1^ Department of Pediatric Surgery, Union Hospital, Tongji Medical College, Huazhong University of Science and Technology, Wuhan, China; ^2^ Key Laboratory of Molecular Biophysics of Ministry of Education, College of Life Science and Technology, Huazhong University of Science and Technology, Wuhan, China; ^3^ China Zebrafish Resource Center, National Aquatic Biological Resource Center, Institute of Hydrobiology, Chinese Academy of Sciences, Wuhan, China

**Keywords:** Hirschsprung’s disease, neuron development, RET, Nrg1, PI3K/AKT

## Abstract

**Introduction:** As a congenital and genetically related disease, many single nucleotide polymorphisms (SNPs) have been reported to be associated with the risk of HSCR. Our previous research showed that SNP rs2439302 (NRG1) interacted with rs2435357 (RET) to increase the risk of HSCR development. However, the underlying molecular mechanism is still not well understood.

**Methods:** SNP rs2439302 (NRG1) and rs2435357 (RET) were genotyped in 470 HSCR cases. The expression of NRG1 and RET was investigated in the colon of HSCR patients. Knockdown of the NRG1 and RET homologs was performed in zebrafish to investigate their synergistic effect on ENS development. The effect of SNP rs2439302 and rs2435357 polymorphism on neuron proliferation, migration, and differentiation were investigated in SHSY-5Y cells and IPSCs.

**Results:** Significant downregulation of NRG1 and RET expression was noticed in the aganglionic segment of HSCR patients and SHSY-5Y cells with rs2439302 GG/rs2435357 TT genotype. NRG1 and RET double mutants caused the most severe reduction in enteric neuron numbers than NRG1 single mutant or RET single mutant in the hindgut of zebrafish. SHSY-5Y cells and IPSCs with rs2439302 GG/rs2435357 TT genotype exhibited a decreased proliferative, migration, and differentiative capacity. CTCF showed a considerably higher binding ability to SNP rs2439302 CC than GG. NRG1 reduction caused a further decrease in SOX10 expression via the PI3K/Akt pathway, which regulates RET expression by directly binding to rs2435357.

**Discussion:** SNP rs2439302 (NRG1) GG increases the risk of developing HSCR by affecting the binding of transcription factor CTCF and interacting with rs2435357 (RET) to regulate RET expression via the PI3K/Akt/SOX10 pathway.

## 1 Introduction

Hirschsprung’s disease (HSCR) is the most common congenital gut motility disorder, with significant contributions from genetic factors. Several genome-wide association studies (GWAS) have discovered hundreds of SNPs associated with increased risk of HSCR, with many associated variants located in the cis-regulatory elements (CREs) of these target genes (Ngan et al., 2011; Jiang et al., 2015; Bae et al., 2016; Chatterjee et al., 2016; Tang et al., 2016; Tang et al., 2018; Tilghman et al., 2019; Chatterjee et al., 2021; Kapoor et al., 2021). Understanding the underlying mechanisms through which they influence enteric nervous system (ENS) development remains an outstanding task for the pathogenesis of HSCR. Recent advances in GWAS and next-generation sequencing (NGS) studies have led to the discovery of a number of new HSCR candidate genes, contributing to approximately 72% of HSCR cases (Gunadi et al., 2023; Tang et al., 2023). *RET* proto-oncogene was the first major HSCR gene discovered in the early 1990s, contributing to 50% of familial and 15%–20% of sporadic HSCR cases (Angrist et al., 1995). Other related genes, including GDNF, EDNRB, SOX10, PHOX2B, NRG1, ERBB2, SEMA3C/D, ITGB4, and BACE2, were discovered subsequently (Karim et al., 2021). However, rare variants of the risk genes cumulatively explained only a modest number of cases, often involving syndromic forms (Jiang et al., 2015). Common non-coding mutations in RET and related genes were more commonly seen.

The representative common functional *RET* intron one enhancer variant (rs2435357 T>C) plays a pivotal role in epistatic interaction within and between HSCR genes (Karim et al., 2021). A whole genome sequencing study that included 443 short-segment HSCR patients and 493 ethnically matched control individuals showed that the strongest association of 328 variants with HSCR were all mapped to the known disease susceptibility loci of *RET* and *NRG1* (Tang et al., 2018). Moreover, several GWAS have shown an epistatic combined effect of SNPs of NRG1 and RET, highlighting the potential interplay between common variants of NRG1 and RET in the genetic etiology of HSCR(Phusantisampan et al., 2012; Gui et al., 2013; GunadiKapoor et al., 2014; GunadiIskandar et al., 2019).

Three common variants, including rs2439302, rs7835688, and rs16879552, were reported to be significantly associated with the risk of HSCR(Tang et al., 2011; Phusantisampan et al., 2012). However, our previous study only found SNPs rs2439302 and rs7835688 showed a positive correlation with HSCR susceptibility and are in high linkage disequilibrium in the central China population. Therefore, in the current study, we report rs2439302 as representative of the association (Yang et al., 2017). *NRG1*/*ERBB3* signaling is essential for Schwann cell proliferation, migration, and myelination in mouse and zebrafish models (Meyer and Birchmeier, 1995; Britsch et al., 1998; Morris et al., 1999; Garratt et al., 2000; Lyons et al., 2005; Perlin et al., 2011; Glenn and Talbot, 2013; Heermann and Schwab, 2013). A previous study has shown that the HSCR risk variants of *NRG1* in the coding region could lead to the downregulation of *NRG1* protein expression in COS-7 cells (Luzón-Toro et al., 2012). In contrast, another research has shown that *NRG1* mRNA and protein expression in both HSCR stenotic and dilated segment samples was significantly higher than in the controls (Tang et al., 2012). Moreover, *NRG1* was reported to favor gliogenesis, and low *NRG1* levels favor neurogenesis (Gui et al., 2013). A systematic understanding of the mutual regulation mechanism of *NRG1* and *RET* is still lacking.

To discover the interplay mechanism of *NRG1* and *RET* common variants in HSCR, we exploited human specimens, zebrafish, SHSY-5Y cell lines, and induced pluripotent stem cells (IPSCs). Our results demonstrated that SNP rs2439302 (*NRG1*) GG increases the risk of developing HSCR by affecting the binding of transcription factor CTCF and interacting with rs2435357 (*RET*) to regulate *RET* expression via the *SOX10*/*PI3K*/*Akt*/pathway.

## 2 Material and methods

### 2.1 Study populations

A total of 470 unrelated sporadic short-segment HSCR patients were studied, which included 364 males and 106 females with a male: female ratio of 3.43:1. The diagnosis of HSCR was based on histological examination of biopsy tissues obtained from the stenosis segment during operations: the absence of ganglion cells and neural hypertrophy. The colon of anorectal malformation patients with colostomy (non-HSCR) were used as the control group. This study was approved by the Research Ethics Board of Union Hospital (No. 2018-S180), Tongji Medical College, Huazhong University of Science and Technology. Informed written consent was obtained from legal guardians of all participants.

### 2.2 Genomic DNA extraction and single-nucleotide polymorphism genotyping

A 2 mL sample of peripheral blood was collected from each included patient. Genomic DNA was extracted from the peripheral blood using a QIAamp DNA Blood Midi Kit (51183, Qiagen, Germany) according to the manufacturer’s protocols. DNA concentrations were determined with Nano Drop 2,000 (Thermo Fisher Scientific, United States), and the samples were stored at −80°C until use. DNA sequences containing the polymorphism rs2439302 (NRG1) and rs2435357 (RET) were amplified and sequenced using the Sanger method. The primers used for amplification and sequencing are listed in Supplementary Table S1.

### 2.3 Immunochemistry staining

Full-thickness ganglionic and aganglionic colon tissues were obtained from patients pathologically confirmed to have HSCR and fixed in 4% paraformaldehyde. After a standard dehydration procedure, the colon tissues were embedded in paraffin blocks and cut into 0.4 μm sections. EDTA was used as an antigen retrieval buffer of NRG1 and RET. S100 retrieval was performed in citric acid sodium citrate buffer. The dilution ratio of primary antibodies was 1:500 for NRG1 and RET and 1:50 for SOX10. The detailed preparation and staining procedures were described in our previous study (27).

### 2.4 Gene expression assays

Total RNA was extracted from frozen HSCR colon tissues (aganglionic, transition, and dilated segments, *n* = 11) or cells (*n* = 3) using TRIzol Reagent (Invitrogen, United States) according to the manufacturer’s instructions. The quality and concentration of total RNA were checked by NanoDrop 2000 (Thermo Fisher Scientific, United States) and gel electrophoresis. For mRNA detection, 1 μg of total RNA was reverse transcribed to cDNA using the RevertAid First Strand cDNA Synthesis kit (K1621, Thermo Scientific, United States). The expression levels of the NRG1 and RET genes were measured by qPCR using the CFX Connect™ Real-Time PCR Detection System (Bio-Rad, United States). The GAPDH gene was chosen as the reference gene. The relative expression level of mRNA was quantified using the ΔΔCt method. The primers are listed in Supplementary Table S1.

### 2.5 Western blotting

Total protein was extracted from HSCR colon tissues (aganglionic, transition, and dilated segments, *n* = 3) or cells (*n* = 3) with a radio-immunoprecipitation assay (RIPA) buffer containing Protease and Phosphatase Inhibitor Cocktail (Halt, Thermo Fisher Scientific, United States). Cell lysates were sonicated, protein concentrations were quantified using the BCA method (P0012S, Beyotime, China), and equivalent protein were resolved by 8% SDS-PAGE (G2061-50T, servicebio, China) in reducing conditions and blotted onto a polyvinylidene fluoride (PVDF) membrane (Roche, Basel, Switzerland). Membranes were blocked using 5% Bovine albumin and incubated with the appropriate diluted antibodies (anti-NRG1, 1:1000, ab191139; anti-RET, 1:1000, ab134100; anti-SOX10, 1:1000, ab227680; Abcam, United Kingdom), (anti-ERBB3, 1:1000, 12708, Anti-Akt, 1:1000, 4,691, anti-pAkt, 1:1000, 4,060, Cell signaling technology, United States), (anti-GAPDH, 1:5000, 60004-1-Ig, HRP-conjugated Affinipure Goat Anti-Mouse IgG (H + L), 1:5000, SA00001-1, HRP-conjugated Affinipure Goat Anti-Rabbit IgG (H + L), 1:5000, SA00001-2, proteintech, China). The protein expression levels were normalized to those of GAPDH. Results are representative of at least three independent experiments.

### 2.6 SHSY5Y cells culture and differential assay

SH-SY5Y cells were cultured and maintained in a 1:1 mixture of Eagle’s Minimum Essential Medium (21090055, Gibco, United States) and F12 Medium (11765054, Gibco, United States) containing 10% FBS (10100147, Gibco, United States) and 1% penicillin/streptomycin in a humidified atmosphere of 5% CO2 at 37°C. The SH-SY5Y cells were differentiated for 6 days by adding all-trans-retinoic acid (ATRA) (10uM) (n = 3). 1 mg/mL ERBB3 antibody (MAB3481 neutralization antibody, R&D, United States) was added to the medium for intervention experiments.

### 2.7 Induced pluripotent stem cell culture

Human induced pluripotent stem cells (IPSCs) from bone marrow-derived mesenchymal stem cells were kindly provided by Dr. Jiaoya Xi. Briefly, IPSCs were cultured and maintained in mTeSR-1 medium (Stemcell Technologies, Canada) with 1% penicillin/streptomycin in a humidified atmosphere of 5% CO2 at 37°C. The medium was changed every day.

### 2.8 Generation of rs2435357 CC and rs2439302 CC cell lines

Cells with the RET rs2435357T>C and NRG1 rs2439302G>C mutations were generated using CRISPR gene editing technology. Based on the genomic sequence of human RET (Gene ID 5979) and NRG1(Gene ID 3084), gRNA was designed with the sequence “CTG​CAG​CCA​AGG​GGG​CCA​GTG​ACC​CTT​ACA​TGG​TCA​CCA​CA” and “TGT​AAT​CTT​TGT​TTC​ATA​GAG​TTT​ACA​CTA​CAG​CTT​TGC​CAC” targeting the area near the mutation sites using the CRISPR design tool (http://www.e-crisp.org/). For SHSY-5Y cells, RET rs2435357 and NRG1 rs2439302 were targeted and mutated by transient co-transfection of plasmids carrying the gRNA, Cas9, and Oligo ssDNA. The isogenic single clones were generated by the limited dilution method. The picked clones were screened by restriction endonuclease digestion and Sanger DNA sequencing to identify isogenic-modified clones. Mutated IPSCs were generated by Neon Transfection System (Invitrogen, CA, United States) with 1000 V, 40 m, and 1pulse. The transfected cells were then plated on the Matrigel-coated plate, and 24 h later, selection with a culture medium containing 20 μg/mL puromycin would be started. Puromycin-resistant colonies were obtained in 2 weeks and isolated for subsequent DNA sequencing. Finally, the rs2435357 CC, and rs2439302 CC modified cell lines were successfully generated, expanded, and tested as mycoplasma-free for subsequent experiments.

### 2.9 Cell proliferation and migration assay

SH-SY5Y cells were seeded in a 96-well plate at a 20,000 cells/mL, and cell viability was detected by cell counting kit-8 (C0037, Beyotime, China) according to the manufacturer’s instructions at 24, 48, and 72 h. The absorbance of wells was measured at 450 nm, and the corresponding cell number in each well was calculated according to the standard curve. Migratory behaviors of SH-SY5Y cells were measured by manually scratching with a 200 μL sterile pipette tip in six-well plates with confluent monolayer cells. Subsequently, the pictures from the cells were taken after 24 h. Scratch areas of microscope images were analyzed by using ImageJ software. Data were reported as mean ± SEM. The proliferation and migration assays were performed in 1 clone with 3 technical replicates.

### 2.10 Immunofluorescence

The SHSY-5Y cells were fixed in 4% paraformaldehyde for 10 min at room temperature, followed by three times of PBS washing and subsequent treatment with 10% goat serum albumin (50197Z, Thermo Fisher Scientific, United States) in PBS for 1 hour. The primary antibodies included anti-human NSE (1:150; Cell Signaling Technology, United States) and anti-GAPDH (1:200, 60004-1-Ig, proteintech, China). The secondary antibodies were Cy3–conjugated Affinipure Goat Anti-Rabbit IgG (H + L), (1:100, SA00009-2, proteintech, China), and CoraLite488-conjugated Goat Anti-Mouse IgG (H + L), (1:100, SA00013-1, proteintech, China). SHSY-5Y cell nuclei were stained with DAPI (C1005, Beyotime, China). Images were acquired under a fluorescence microscope.

### 2.11 Luciferase assay

To construct the human NRG1 3′UTR luciferase reporter (NRG1-WT) and the CTCF target site-mutation NRG1 3′UTR luciferase reporter (NRG1-Mut), we have the full-length 3′-UTR of NRG1 mRNA and the mutant site at rs2439302 amplified and cloned into pGL3-basic luciferase reporter vector. pcDNA3.1-CTCF overexpression plasmid was constructed and transferred to Competent *E. coli* and plated onto Luria Bertani medium containing ampicillin. After sequencing, the correct colony was selected, and the plasmid was extracted using Plasmid Mini Kit (D6942-01, Omega, United States). Neofect DNA transfection reagent (TF201201, Beijing, China) was used to co-transfect plasmids into Human Embryonic Kidney (HEK) 293 T cells. The luciferase activities were tested by Dual-Luciferase Assay system (Promega) after 24 h’ incubation following the manufacturer’s instructions (*n* = 3).

### 2.12 Derivation of neural crest cells from iPSCs

Differentiation was initiated by adding 0.1 μM LDN193189 and 10 μM SB431542 and gradually switching the mTeSR1 medium with N2 medium, as described previously (28). After 10 days of induction, the cells were resuspended in an N2-differentiation medium containing 200 μM Ascorbic Acid (A4034, Sigma, United States), 20 ng/mL BDNF (DC076, novoprotein, China), 100 ng/mL FGF8 (C798, novoprotein, China), 20 ng/mL SHH (C100, novoprotein, China), 10 μM Y-27632 dihydrochloride (S1049, Selleck, United States) at the concentration of 120,000 cells/10 μL and plated 10 μL droplets close to each other without touching onto the dried PO (Poly-L-ornithine hydrobromide, P3655, Sigma, United States)/Lam (laminin, 354239, BD, United States)/FN (Fibronectin, 356008, BD, United States) 15 cm dishes. On day 18, some cells exhibited neural crest-like morphology and were analyzed for expression of neural crest cell surface markers by flow cytometry.

### 2.13 Flow cytometry analysis

The IPSC cells were dissociated with Accutase for 20–30 min at 37°C and resuspended in PBS. For surface markers detection, cells were incubated for 30 min at room temperature with APC anti-human CD271 (p75NTR) antibody (345107, Biolegend, United States) and PerCP/Cyanine5.5 anti-human CD57 Recombinant Antibody (393311, Biolegend, United States). The labeled cells were detected using a FACSCalibur instrument (*n* = 3). FlowJo version 10.4.0 (Tree Star, Inc., Ashland, OR) was used to analyze the flow data.

### 2.14 Zebrafish maintenance and transgenic lines used

Zebrafish were cultured in a circulated water system at 28.5°C and in a daily cycle of 14-hour-light and 10-hour-dark. NRG1ihb534/+; RETihb309/+ zebrafish (purchased from China zebrafish resource center) was used to analyze how the NRG1 and RET double mutation affect neuron development *in vivo*. Embryos were obtained from adult fish pairs by natural spawning and were raised at 28.5 °C in tank water.

### 2.15 Whole mount immunohistochemistry for zebrafish

Our previous study showed that NRG1 had the highest expression in 3-day zebrafish embryos (Pu et al., 2017). Thus, 72hpf zebrafish embryos were collected for the following experiments. Zebrafish embryos were fixed overnight by 4% PFA at 4°C. After a series of washing, embryos were treated with proteinase K (20 μg/mL) for 30min and blocked with PBDT (PBS with 1%BSA, 1%DMSO, and 0.1% tritonx-100) with 10% goat serum at room temperature for 2 h. The HuC/D antibody (ab210554, Abcam, United States) was stained for ENS neurons at 4°C overnight. ABflo™ 488-conjugated Goat Anti-Rabbit IgG (H + L) antibody (AS053, 1:100, Abclonal, China) was used to incubate the embryos.

### 2.16 Whole mount *in situ* hybridization for zebrafish

Template zebrafish cDNA was obtained from 72°hpf zebrafish embryos. Target fragments of NRG1, RET, and SOX10 were amplified and validated via gel electrophoresis and Sanger sequencing. The primers are shown in Supplementary Table S1. Probes were synthesized and labeled with the DIG RNA Labeling Kit (11175025910, Roche, United States). Zebrafish embryos at 72 hpf were fixed with 4% paraformaldehyde (PFA) overnight at 4°C. Embryos were digested with 10 μg/mL proteinase K for 30 min at room temperature. The embryos were prehybridized for 2 h in a prehybridized solution and then hybridized overnight at 65°C. The signals were detected using alkaline-phosphatase-conjugated anti-DIG antibody (11093274910, Roche, United States) and NBT/BCIP solution (11681451001, Roche, United States) following the manufacturer’s instructions. The embryos were examined and photographed under a light microscope (BX53, Olympus). Quantitively analysis of the fluorescence intensity around the gut (circled in the picture) was measured by analyzing the embryo pictures with ImageJ software. Six embryos were measured in each group.

## 3 Results

### 3.1 Protein–protein interaction network of *NRG1* and *RET*

**FIGURE 1:**
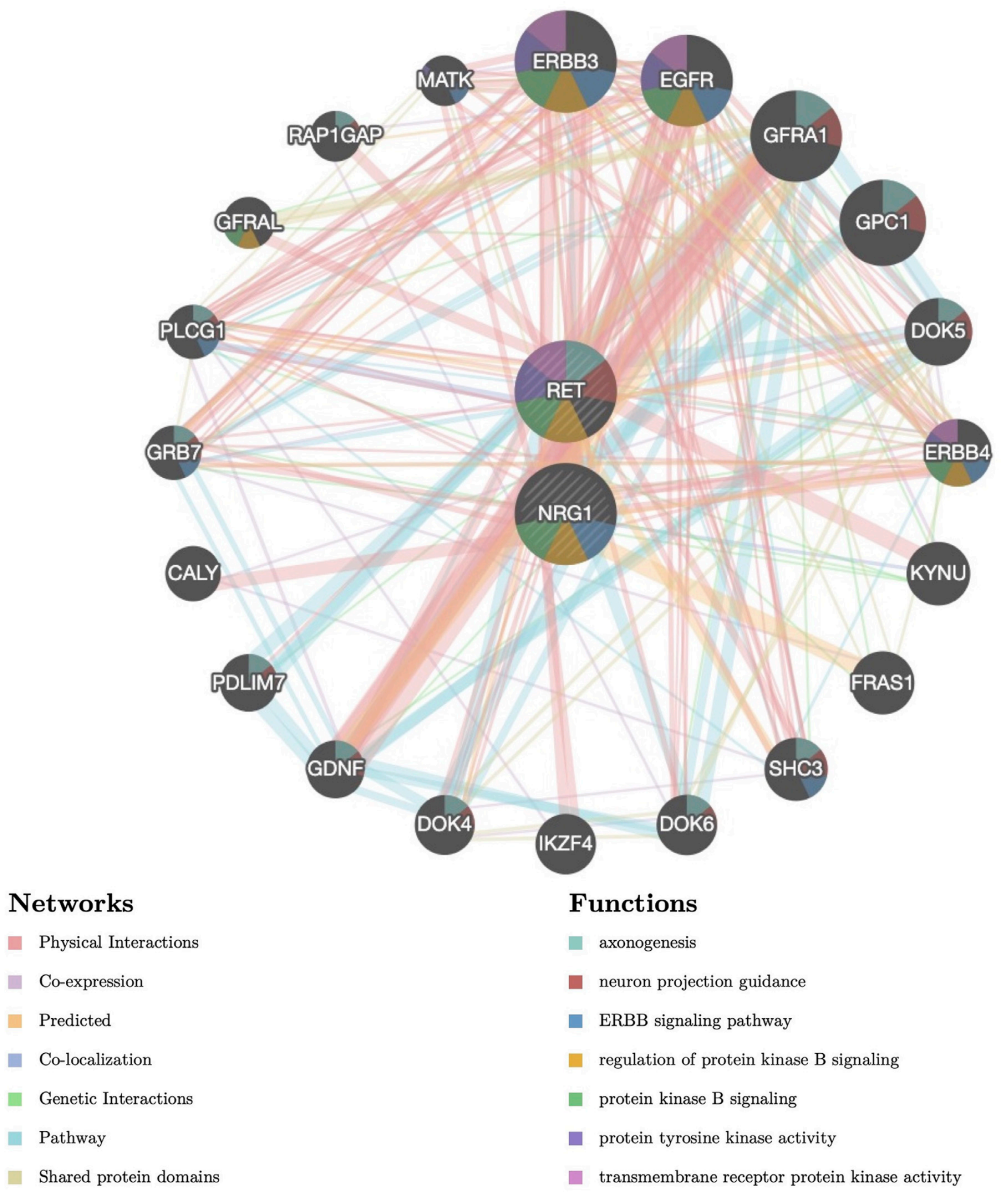
Protein–protein interaction (PPI) network of *NRG1* and *RET*. Proteins and signaling pathways significantly related to *NRG1* and *RET* were illustrated. The size of the surrounded nodes represents the ranking of the relationships. The interconnected lines represent the predicted networks (physical interactions, co-expression, predicted, co-localization, genetic interactions, pathway, and shared protein domains). The color of the surrounded nodes represents the predicted function of the genes (axonogenesis, neuron projection guidance, ERBB signaling pathway, regulation of protein kinase B signaling, protein tyrosine kinase activity, transmembrane receptor protein kinase activity).

We have previously shown that the OR for the NRG1 rs2439302 risk homozygote (GG) was increased by 25.57-fold in the presence of the RET rs2435357 risk genotype (TT), revealing enhanced HSCR risk (Yang et al., 2017). We constructed a protein-protein interaction (PPI) network for the NRG1 and RET with critical signaling pathway genes and evaluated the correlations using the Gene MANIA database. (Figure 1). Functional prediction revealed that NRG1 and RET showed correlations with neuron projection guidance (False Discovery Rate (FDR) = 2.16 × 10^−14^), axonogenesis (FDR = 6.93 × 10^−13^), ERBB signaling pathway (FDR = 6.76 × 10^−9^), and regulation of protein kinase B signaling (FDR = 2.48 × 10^−4^). SNP rs2435357 in intron 1 of *RET* exhibits enhancer activity through the direct binding of SOX10 (Chatterjee et al., 2016). Moreover, the NRG1/ErbB signaling pathway was reported to play an important role in promoting the maintenance of SOX10 (Yang et al., 2022). These results indicated that the ERBB signaling pathway, PI3K/Akt signaling pathway (Protein kinase B signaling pathway), and SOX10 might be involved in interactions between *NRG1* and *RET*.

### 3.2 Polymorphisms distribution of *NRG1* rs2439302 and *RET* rs2435357

To find the rs2439302 GG and rs2435357 TT colon tissue and further verify the previous results, we performed genome sequencing in 470 HSCR patients. SNP rs2439302 with risk allele G (non-risk allele C) and rs2435357 with risk allele T (non-risk allele C) have allele frequencies of 0.29 and 0.73 in cases against a background allele frequency of 0.19 and 0.50 in our previous published normal control cohort. Among the 470 included HSCR patients, 29 (6.17%) patients have genotype rs2439302 GG and rs2435357 TT and the corresponding colon tissues were used for subsequent experiments. The data can be accessed at https://www.ncbi.nlm.nih.gov/sra/PRJNA953977.

### 3.3 Expression pattern of NRG1, RET, and SOX10 in HSCR and control colon

**FIGURE 2:**
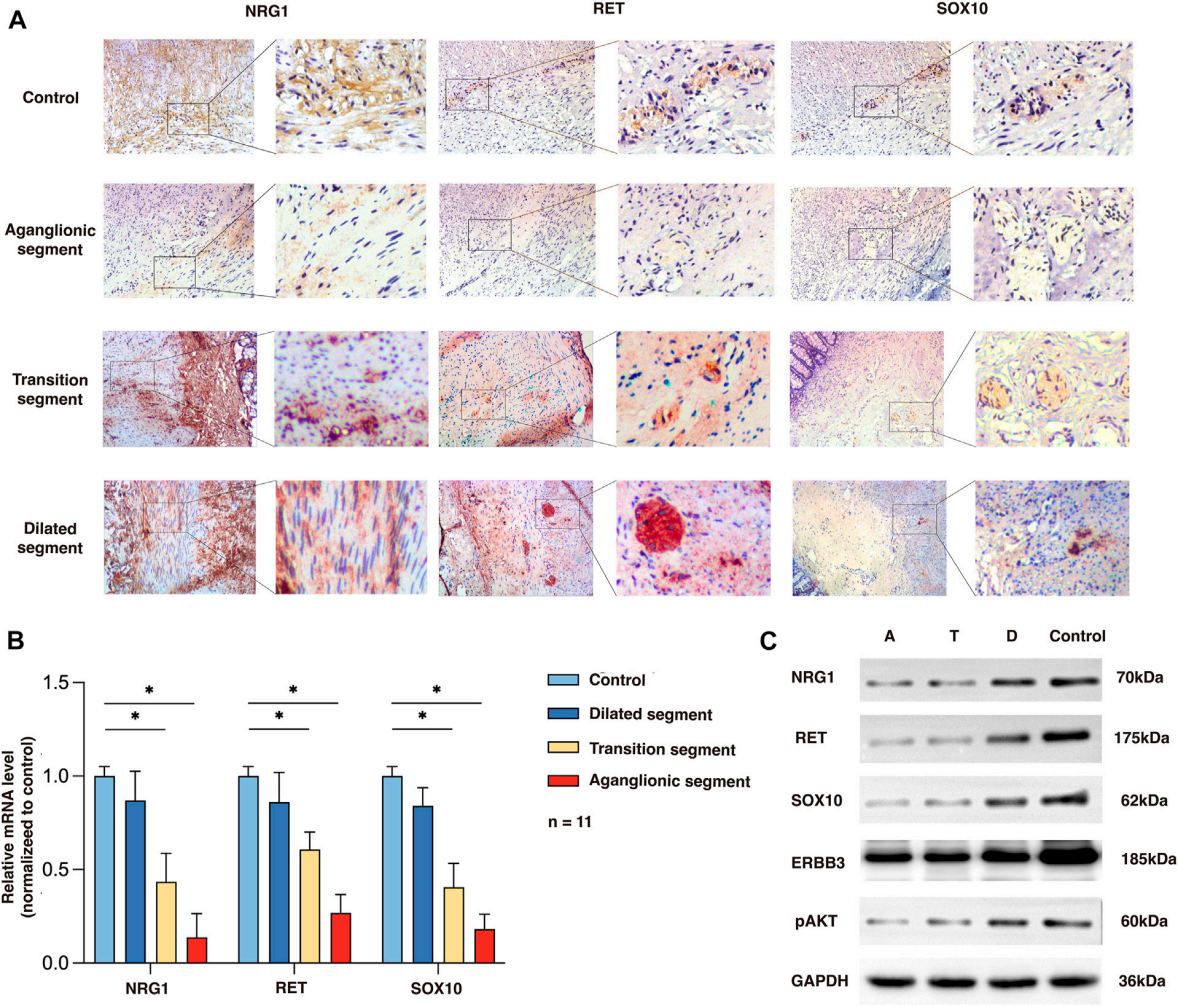
Expression of NRG1, RET, and SOX10 in the rs2439302 GG rs2435357 TT HSCR patients’ colon and control colon. **(A)** Immunochemistry staining in colon tissues for NRG1, RET, and SOX10. **(B)** qRT-PCR analysis of relative expression levels of NRG1, RET, and SOX10. **(C)** Western blot of protein expression levels of NRG1, RET, SOX10, ERBB3, pAKT, and GAPDH in aganglionic, transition, and dilated segments of HSCR patients and control.

To determine the expression and location of NRG1, RET, and SOX10 in the human colon, we performed immunochemistry in the aganglionic HSCR segments (rs2439302 GG/rs2435357 TT) as well as in colon tissue of non-HSCR control. NRG1 expression in HSCR patients (rs2439302 GG/rs2435357 TT) was significantly reduced in the muscle layer of the aganglionic and transition segments than in normal controls. *RET* and *SOX10* were mainly expressed in the myenteric plexus in the normal colon, while no obvious expression was seen in aganglionic HSCR segments. A small amount of RET and SOX10 expression could be seen in transition segments. The NRG1, RET, and SOX10 expressions in dilated segments were close to normal controls. (Figure 2A). We performed qRT-PCR and Western blot analysis on aganglionic, transition, and dilated HSCR segments (rs2439302 GG/rs2435357 TT genotype) and in colon tissue of non-HSCR control. *NRG1*, *RET*, and *SOX10* were found to be downregulated in HSCR segments. (Figures 2B, C).

### 3.4 NRG1 and RET zebrafish mutant has developmental defects in neural crest derivatives

**FIGURE 3:**
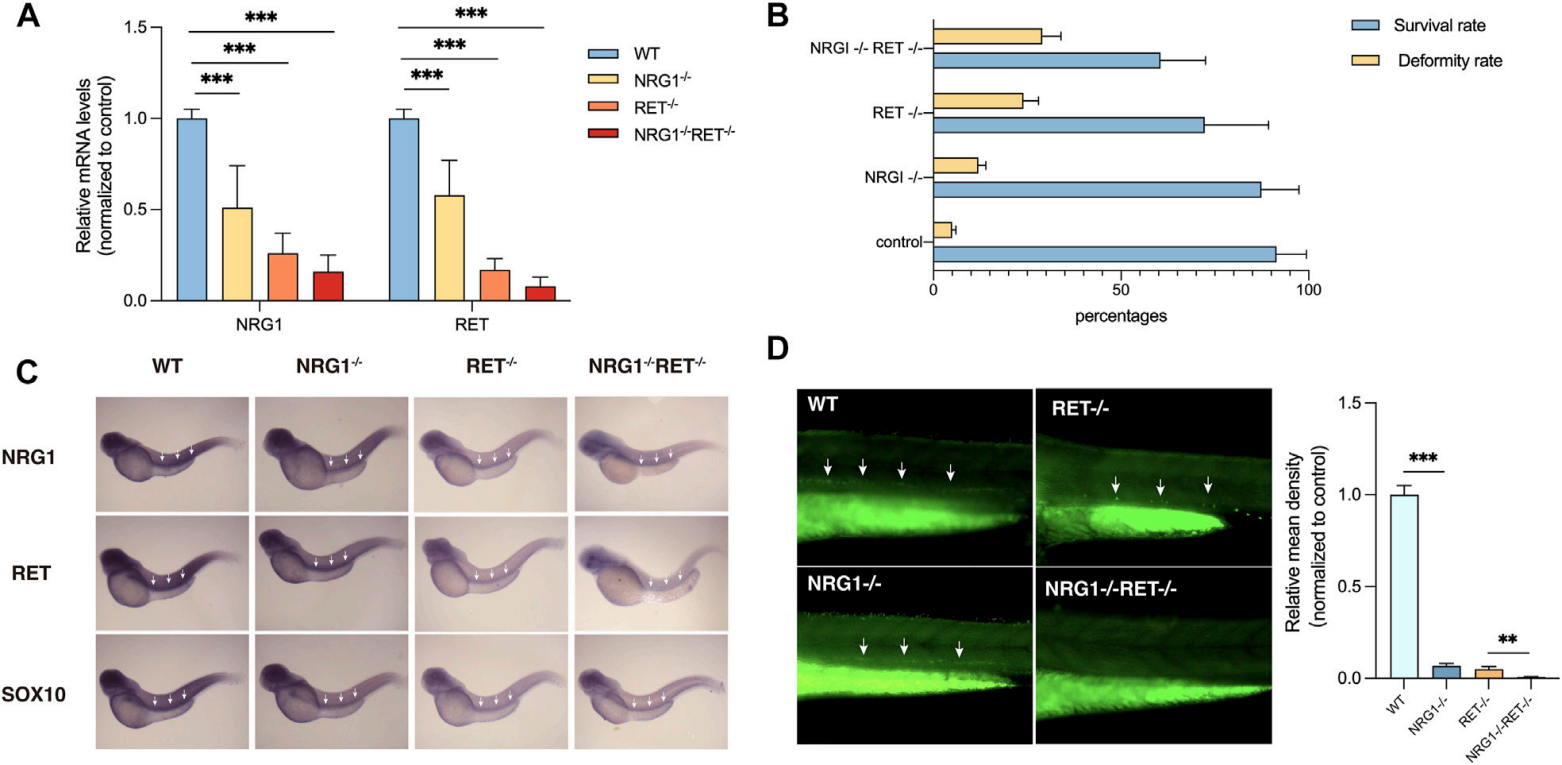
Phenotypes of nrg1 and ret knockdown zebrafish. **(A)** qRT-PCR analysis of relative expression levels of nrg1 and ret in wildtype, *nrg1* ^−/−^, *ret* ^−/−^, and *nrg1* ^−/−^ *ret* ^−/−^ zebrafish embryos at 72 hpf. **(B)** The survival and deformity rate of wildtype, *nrg1* ^−/−^, *ret* ^−/−^, and *nrg1* ^−/−^ *ret* ^−/−^ zebrafish at 72 hpf. **(C)** Whole-mount *in situ* hybridization in wildtype, *nrg1* ^−/−^, *ret* ^−/−^, and *nrg1* ^−/−^ *ret* ^−/−^ embryos at 72 hpf for *nrg1*, *ret*, and *sox10*. White arrows indicated the end of the positive expression of each gene. **(D)** Immunofluorescence staining and quantitative analysis of a neuronal marker HuC/D in wildtype, *nrg1* ^−/−^, *ret* ^−/−^, and *nrg1* ^−/−^ *ret* ^−/−^ embryos at 72 hpf.

*NRG1* and *RET* were found to be downregulated in colon tissues of rs2439302 GG/rs2435357 TT genotype HSCR patients. To verify whether the simultaneously decreased *NRG1* and *RET* expression levels would affect enteric neurons development, we generated a −22bp +5 bp alteration in the exon 3 of the zebrafish *nrg1* gene and a -5 bp deletion in the exon 3 of the zebrafish *ret* gene using CRISPR/Cas9, predicted to generate a premature stop codon. Zebrafish *nrg1* has five protein-coding transcripts and ten exons, making INDELS at exon 3 of nrg1 only interfere with four transcripts. Transcript nrg1-206 was not affected. The RT-PCR results confirmed the partial knockdown instead of the knock-off of *nrg1* and *ret*. (Figure 3A). *nrg1* ^−/−^ *ret* ^+/+^ and *nrg1* ^−/−^ *ret* ^−/−^ fish resulted in morphological defects, mainly including pericardial effusion and shortened body axis in a proportion of embryos. The survival rates were 61% (66/108), 72% (116/161), 87% (126/145), and 91% (320/352) in *nrg1* ^−/−^ *ret* ^−/−^, *nrg1* ^−/−^ *ret* ^+/+^, *nrg1* ^−/−^ *ret* ^+/+^, and WT groups. *nrg1* ^−/−^ *ret* ^−/−^ double mutation fish had a significantly higher proportion of embryos with morphological defects. (Figure 3B).

*In situ* hybridization results showed that *nrg1*, *ret*, and *sox10* mRNA expression levels were reduced in *nrg1* ^−/−^, *ret* ^−/−^, and *nrg1* ^−/−^ *ret* ^−/−^ embryos. (Figure 3C). Immunostaining of a zebrafish with a neuronal marker, *HuC/D*, showed that the density of enteric neurons was significantly reduced in the *nrg1* ^−/−^ *ret* ^−/−^ double mutation embryos compared with *nrg1* ^−/−^ single mutation embryos, *ret* ^−/−^ single mutation embryos, and control sibling embryos. (Figure 3D).

### 3.5 rs2439302 GG/rs2435357 TT genotype SHSY-5Y cells and IPS-ENCCs display decreased proliferative, migration and differentiative capacity

We showed that simultaneously decreased *NRG1* and *RET* expression could lead to ENS developmental defects. Back to the SNP level, to elucidate how the rs2439302/rs2435357 variants interrupt the ENS development and eventually lead to HSCR disease, we generated rs2439302 CC/rs2435357 TT and rs2439302 CC/rs2435357 CC SHSY-5Y cells and human induced pluripotent stem cells (hiPSC) using CRISPR/Cas9. ENCCs were generated from hiPSC, modeling the progressive differentiation processes during ENS development.

**FIGURE 4:**
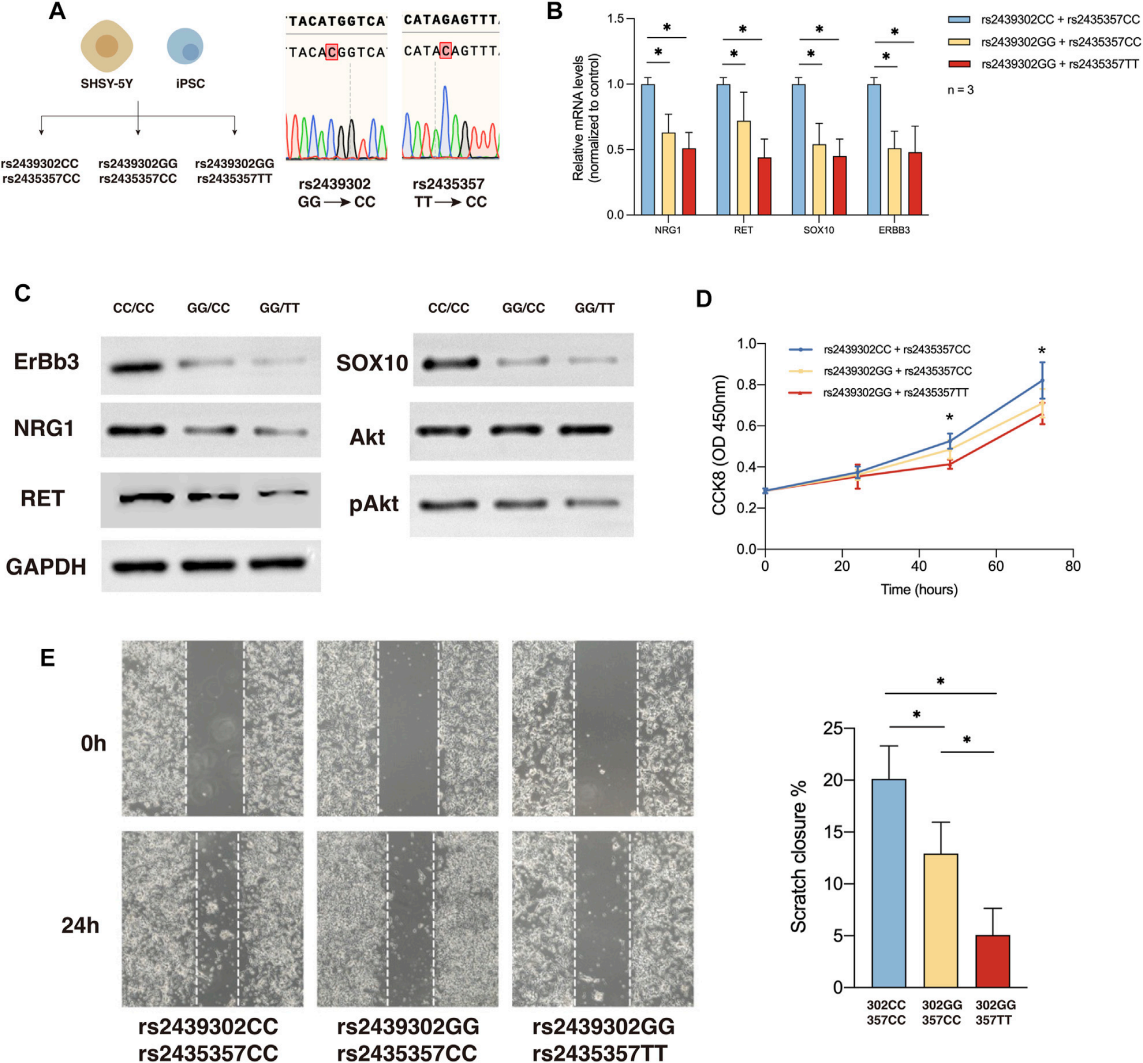
NRG1, RET, SOX10 expression, migration and proliferation ability of SHSY-5Y cells with different genotype. **(A)** Generation of rs2439302 CC/rs2435357 CC and rs2439302 GG/rs2435357 CC cell lines and confirmation by sanger sequencing. **(B)** qRT-PCR analysis of relative expression levels of NRG1, RET, SOX10, and ERBB3 in SHSY-5Y cells. **(C)** Western blot of protein expression levels of NRG1, RET, SOX10, ERBB3, Akt, pAkt, and GAPDH in SHSY-5Y cells. **(D)** Scratch assays showed that rs2439302 CC/rs2435357 CC SHSY-5Y cells retained the smallest scratch area. **(E)** CCK8 assay results of SHSY-5Y cells with different genotypes.

QRT-PCR and Western blotting of SHSY-5Y cells showed *NRG1*, *RET*, *SOX10*, and *ERBB3*, and p*Akt* expression in rs2439302 CC/rs2435357 TT, rs2439302 GG/rs2435357 TT SHSY-5Y cells decreased compared with rs2439302 CC/rs2435357 CC SHSY-5Y cells. (Figures 4B, C). The scratch assay and CCK8 assay showed that the rs2439302 GG/rs2435357 TT SHSY-5Y cells had a reduced ability to migrate and proliferate. (Figures 4D, E).

**FIGURE 5:**
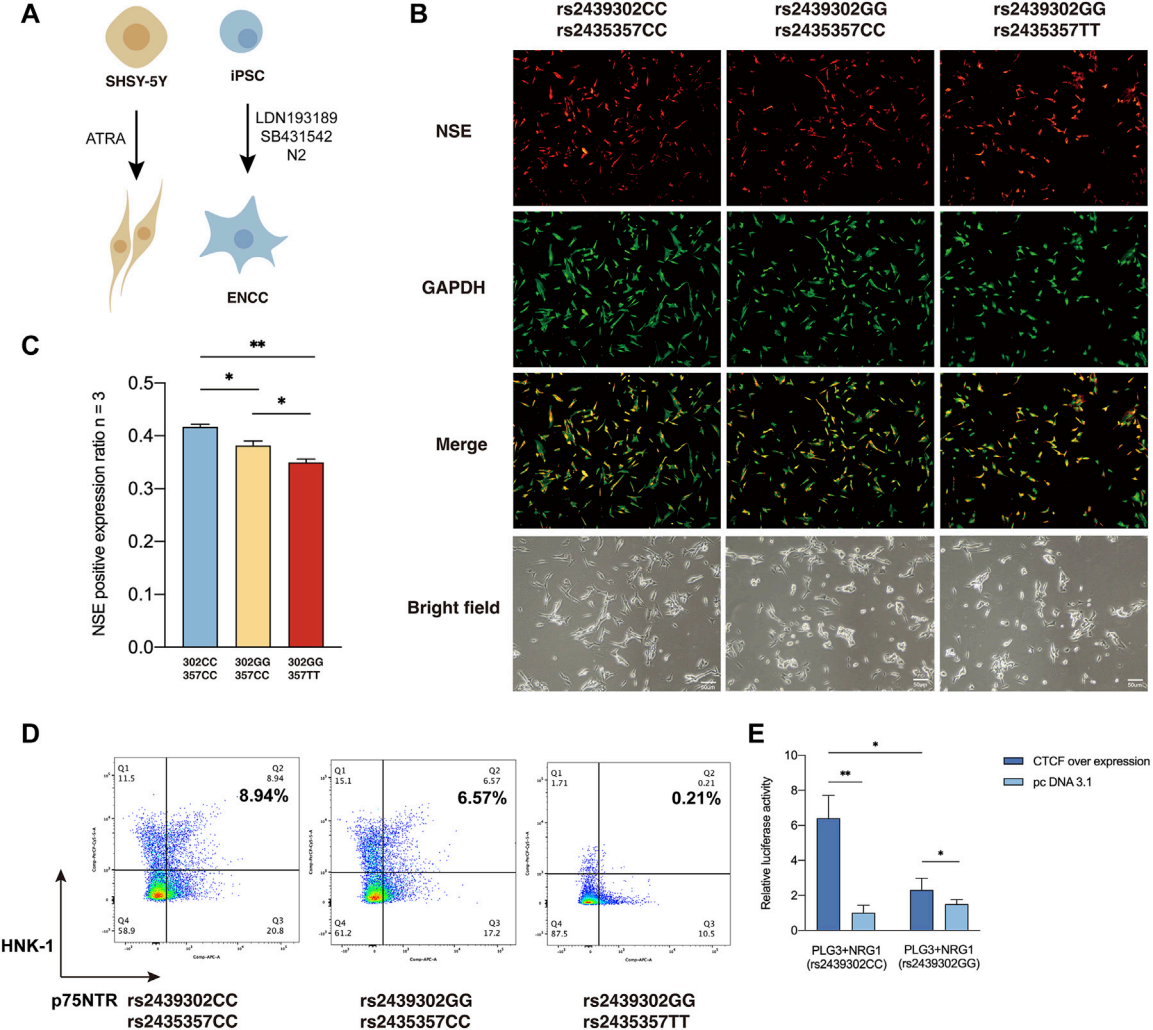
Differentiation ability of SHSY-5Y cells and IPSCs with different genotype. **(A)** Differentiation of SHSY-5Y cells were induced by ATRA and ENCCs were obtained from IPSCs by adding LDN193189 and SB431542 to the differentiation medium. **(B)** Immunofluorescence staining of a SHSY-5Y differentiation marker NSE and bright field morphology of SHSY-5Y cells with different genotypes. **(C)** Semi-quantitative analysis of a SHSY-5Y differentiation marker NSE in SHSY-5Y cells with different genotypes. **(D)** Flow cytometry analysis of the ENCC differentiation rate (HNK-1+ p75NTR+) of IPSCs with different genotypes. **(E)** Luciferase assay showed that CTCF had significantly higher binding ability to SNP rs2439302 CC than GG in SHSY-5Y cells.

We then examined how rs2439302/rs2435357 variations interfere with the responsiveness of SHSY-5Y cells to all-trans-retinoic acid (ATRA) based on their neuronal differentiation capability (Pezzini et al., 2017; Zhang et al., 2021; Al-Maswary et al., 2022), as monitored by the expression of neuron-specific enolase (*NSE*) (Figure 5A). In the presence of ATRA, robust neuronal differentiation was observed in rs2439302 CC/rs2435357 CC SHSY-5Y cells. Rs2439302 CC/rs2435357 TT SHSY-5Y cells differentiated into neurons less efficiently, while rs2439302 GG/rs2435357 TT genotype had a much more significant inhibition effect on the differentiation procedure. (Figures 5B, C). We also examined the peripheral neuronal differentiative ability of iPSCs with different rs2439302/rs2435357 genotypes (Figure 5A). A comparison of the expression level of *HNK-1* and *p75NTR* double-positive (ENCC markers) showed that rs2439302 GG/rs2435357 TT iPSCs had the lowest peripheral neuronal differentiative ability. (Figure 5D). The ENCC differentiation ratio was lower than previous reports, which could be due to the different IPSC origins (bone marrow mesenchymal stem cells and fibroblast) (Zeltner et al., 2014; Lai et al., 2017).

### 3.6 CTCF shows more significant upregulation of NRG1 expression in rs2439302 CC genotype

CCTC-Binding Factor (*CTCF*) plays an essential role in architectural proteins, particularly neurodevelopment (Cummings and Rowley, 2022). Previous studies showed that *CTCF* participates in the maintenance and survival of neuroprogenitor cells (Watson et al., 2014). TFBS Chip-seq data from the UCSC genome browser showed that rs2439302 was located within the *CTCF* binding region in cell lines GM12878, AG09319, and HCT116 (Wang et al., 2013). Using luciferase reporter assay, we tested the function of *NRG1* rs2439302 in the human neuroblastoma cell line SHSY-5Y over expression CTCF. Importantly, amplicons harboring the GG allele have significantly lower enhancer activity than those containing the CC allele. (Figure 5E).

### 3.7 Inhibition of ERBB3 resulted in significantly reduction in the expression level of NRG1 and RET in rs2439302 GG/rs2435357 TT genotype SHSY-5Y cells

**FIGURE 6:**
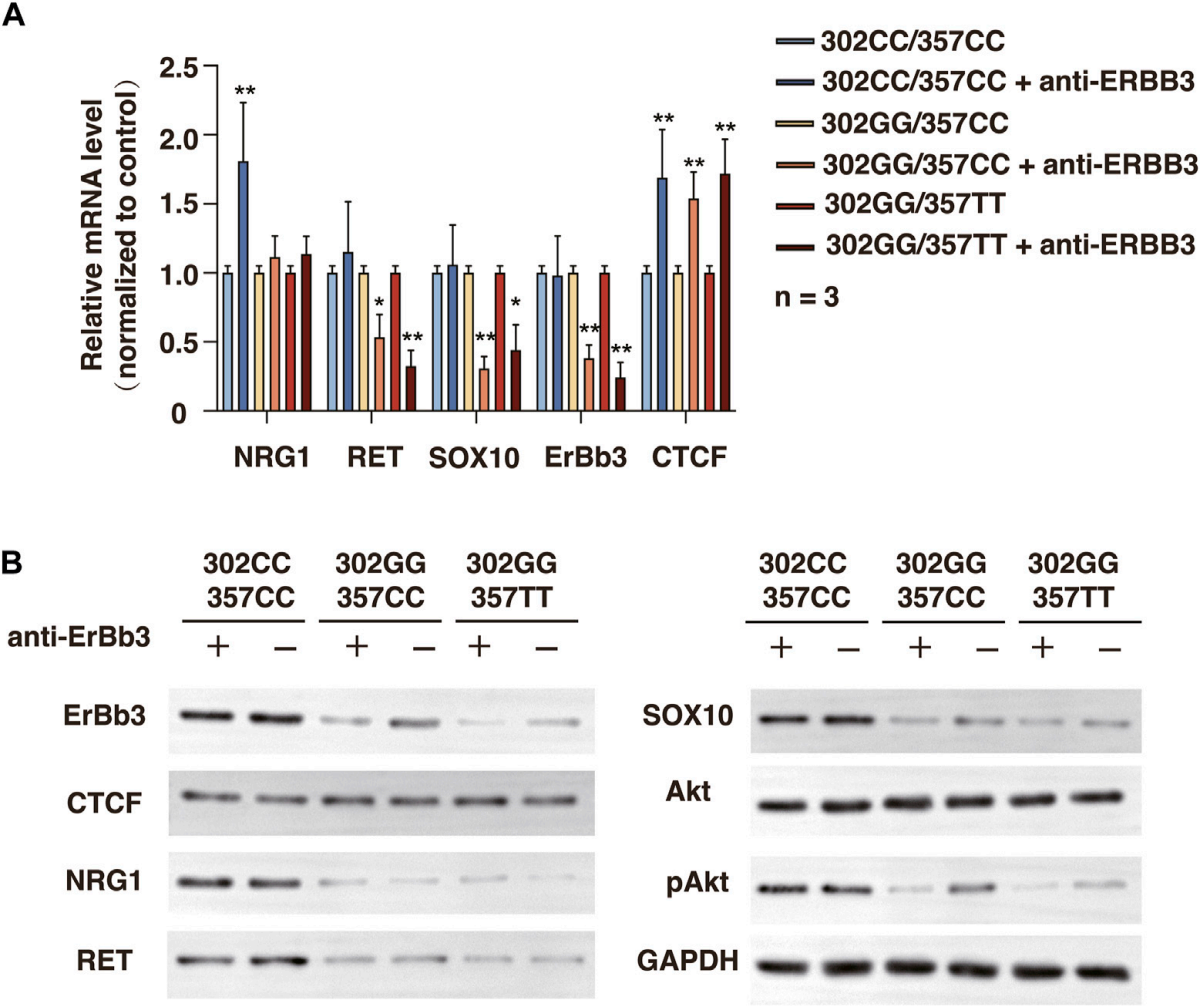
Related protein expression changes after ERBB3 neutralization antibody intervention in SHSY-5Y cells with different genotype. **(A)** qRT-PCR analysis of relative expression levels of NRG1, RET, SOX10, ERBB3, and CTCF. **(B)** Western blot of protein expression levels of NRG1, RET, SOX10, ERBB3, CTCF, Akt, pAkt, and GAPDH.

We next investigate the outcome of ERBB3 selective inhibition by ERBB3 antibody in SHSY-5Y cells. Treatment of ERBB3 antibody over 3 days resulted in a significantly decreased RET and SOX10 expression and Akt phosphorylation level in rs2439302 GG SHSY-5Y cells, while no significant differences were noted in rs2439302 CC SHSY-5Y cells (Figure 6). Interestingly, the NRG1 and ERBB3 expression levels were increased in rs2439302 CC SHSY-5Y cells.

Overall, these data indicated that the different binding tendencies of CTCF with rs2439302 at the enhancer region of *NRG1* could regulate *NRG1* expression and display signs of SOX10, PI3K/Akt pathway, and RET downregulation.

## 4 Discussion

Here we report the possible mechanism of rs2439302 GG/rs2435357 TT genotype patients exhibited a higher penetrance of HSCR. *In vitro* studies showed that *NRG1* rs2439302 GG and *RET* rs2435357 TT could lead to a significant downregulation of NRG1 and RET protein expressions and phosphorylation of the Akt. The interplay between *NRG1* and *RET* regulates *SOX10* and *PI3K*-*Akt* pathway, which further controls nerve cell proliferation, migration, and differentiation.

Identification of genetic markers of susceptibility to Hirschsprung’s disease prompted the investigation of their association with pathogenesis and exploring treatment options (Karim et al., 2021). The joint effect of *NRG1* and *RET* common variants in Asian population have been revealed by several epistatic studies (Phusantisampan et al., 2012; Gui et al., 2013; GunadiKapoor et al., 2014; GunadiIskandar et al., 2019). However, *NRG1* SNP polymorphism did not seem to matter in most European HSCR cases and no joint effect with *RET* SNP polymorphism was found (Kapoor et al., 2015; Fadista et al., 2018; Kapoor et al., 2021). These st (Salerno et al., 2020)udies suggested that *NRG1* SNP polymorphism may have an Asian-specific pathogenic pattern. Our previous case-control analysis in Chinese population found that common variants of *NRG1* rs2439302 confer altered risk to HSCR, and the genetic interaction between *NRG1* rs2439302 and *RET* rs2435357 significantly enhanced the risk of HSCR by 25.57-fold (Yang et al., 2017). In this study, we tried to clarify the underlying molecular mechanism.

SNP rs2439302 was within the first intron of *NRG1* on 8p12, which was first described to be significantly correlated with thyroid cancer (Guo et al., 2022). Thyroid hormones are important regulators of neurodevelopment early in life (Leung and Leung, 2019; Salerno et al., 2020). A recent cross-sectional study showed that thyroid dysfunction was found in approximately 29% of HSCR patients (Xie et al., 2023). Whether *NRG1* rs2439302 polymorphism was associated with the risk of HSCR with thyroid dysfunction or persistent post-operative constipation needs further study. In HSCR, rs2439302 polymorphism was reported to have susceptibility to non-syndromic HSCR in the Chinese population, while similar results were not seen in an Iran cohort (Hosseini-Jangjou et al., 2021). This inconsistency may be due to the different allele frequencies of rs2439302 in different races. The role of rs2439302 polymorphism in *NRG1* expression regulation is controversial as well. In thyroid tissue, Huiling et al. reported that the allele G is associated with the upregulation of *NRG1* (He et al., 2018). However, Rogounovitch et al. found an *NRG1* downregulation in thyroid cancer patients with rs2439302 allele G (Rogounovitch et al., 2015). Our previous study in HSCR patients showed that *NRG1* rs2439302 G was associated with a downregulation of *NRG1* expression (Yang et al., 2017). In the current study, we found in the aganglionic colon of HSCR patients with rs2439302 GG and rs2435357 TT, *NRG1* expression was significantly lower than that in the transition segment, dilated segment, and normal control colon. Bioinformatic functional analysis of SNP rs2439302 showed that it was located within the *CTCF* binding region in cell lines GM12878, AG09319, and HCT116 (Wang et al., 2013). Our current result found that the risk allele of rs2439302 G was shown to disrupt the *CTCF* transcription factor’s binding site in the *NRG1* enhancer in SHSY-5Y cells. Thus, a significant difference in *NRG1* expression was found between CC and GG homozygotes patients and SHSY-5Y cells.

In the literature, *NRG1* was reported to play a vital role in the development and maintenance of the enteric nervous system in several studies (Shi and Bergson, 2020). The interplay of NRG1 and RET is still inconclusive, both synergic and antagonism effect have been reported. In GDNF/RET-induced enteric neural crest cells differentiation, exogenous *NRG1* could reduce the differentiation efficiency and GDNF/RET could negatively regulated NRG1-signaling by down-regulating the expression of its receptor, ERBB (Garcia-Barcelo et al., 2009; Gui et al., 2013). However, in another study, *RET* was reported to specifically increase the expression level of NRG1-Ig isoform (Fleming et al., 2016), which does not have the ERBB receptor binding EGF domain, and thus could not activate the downstream ERBB(Perlin et al., 2011). Moreover, research on HSCR reported significantly upregulated *NRG1* expressions in patients with HSCR compared with control colons (Tang et al., 2012; Gunadi et al., 2022). However, we have not observed increased NRG1 levels in HSCR patients or a neural cell line (SHSY-5Y) with the rs2439302 GG genotype. Interestingly, in HSCR patients with rs2439302 GG and rs 2435357 TT genotype, *NRG1* and *RET* expression were downregulated synergistically. Moreover, *RET* and *NRG1* double mutation zebrafish embryos showed the most severe neurodevelopmental disorders compared to single mutation and control sibling embryos. This rather contradictory result may be due to the complex interaction between *RET* and *NRG1*.

Although no related research studies have shown that *NRG1* could directly regulate *RET* expression, the mutual regulation between *SOX10* and *NRG1* in neuronal development has been confirmed (Van Ho et al., 2011; Zhang et al., 2015; Shea et al., 2020; Yang et al., 2022). SOX10 is a vital transcription factor of neurogenesis and neural crest development, which controls stemness, cell fate, and differentiation (Pingault et al., 2022). Recently, downregulation of SOX10 was reported to play an important role in the development of HSCR(Huang et al., 2022). Both *RET* and *NRG1* were reported to interact with *SOX10*. NRG1/ERBB3 signaling pathway plays a vital role in the differentiation of bone marrow mesenchymal stem cells into Schwann-like cells and can promote the maintenance of SOX10 (Yang et al., 2022). *SOX10* could regulate melanoma growth via *PI3K*/*Akt* pathway activation (Zheng et al., 2018). And Akt activation could promote SOX10 expression as well (Ciarlo et al., 2017), which indicted there could be a two-way regulation between SOX10 and PI3K/Akt pathway. Moreover, SOX10 was reported to regulate *RET* expression by directly binding to the enhancer region around SNP rs2435357 (Emison et al., 2005). The present study also showed that the changes in *SOX10* expression level followed the same trend of *NRG1*, *ERBB3*, *RET*, and *PI3K*/*Akt* pathways.

An equivalent dose intervention of ERBB3 neutralization antibody could lead to different outcomes in SHSY-5Y cells with different rs2439302/rs2435357 genotypes. We noticed a slight increase in CTCF expression after inhibiting the function of ERBB3. Previous studies showed that CTCF plays a key role during neurodevelopment, and CTCF is also a transcript regulator of ERBB3 (Chen et al., 2019; Cummings and Rowley, 2022). NRG1 could also regulate the expression of ERBB3 through the ERBB3/NRG1 autocrine loop (Sheng et al., 2010). Thus, the ERBB3 neutralization antibody groups showed lower ERBB3 levels. The reduced NRG1/ERBB3 pathway activation would lead to difficulty maintaining a stable, effective concentration of SOX10 in the cell. Moreover, combined with the rs2435357 TT genotype, which had a weaker binding ability with SOX10, there would be a significantly reduced RET expression level.

These finding suggested that the interplay between *NRG1* rs2439302 and *RET* rs2435357 is mediated by SOX10. However, we are not able to show the direct molecular regulatory mechanism between SOX10 and PI3K/Akt in the current study. The association between *CTCF*, *NRG1*/*ERBB3*, *PI3K*/*Akt*, *SOX10*, and *RET* obtained in the present investigation implies the underlying mechanism that patients with rs2439302 GG/rs2435357 TT genotype are at higher risk of developing HSCR.

In conclusion, this study demonstrates that common genetic variants, rs2439302 (*NRG1*) GG increase the risk of developing HSCR by affecting the binding of transcription factor *CTCF* and interacting with rs2435357 (*RET*) via *SOX10*/*PI3K*/*Akt* pathway and provided further evidence in the relationship of *NRG1* abnormal expression with HSCR pathogenesis.

## Data Availability

The datasets presented in this study can be found in online repositories. The names of the repository/repositories and accession number(s) can be found below: https://www.ncbi.nlm.nih.gov/SRA at PRJNA953977.

## References

[B1] Al-Maswary A. A. O'Reilly M. Holmes A. P. Walmsley A. D. Cooper P. R. Scheven B. A. (2022). Exploring the neurogenic differentiation of human dental pulp stem cells. PLoS One 17 (11), e0277134. 10.1371/journal.pone.0277134 36331951 PMC9635714

[B2] Angrist M. Bolk S. Thiel B. Puffenberger E. G. Hofstra R. M. Buys C. H. (1995). Mutation analysis of the RET receptor tyrosine kinase in Hirschsprung disease. Hum. Mol. Genet. 4 (5), 821–830. 10.1093/hmg/4.5.821 7633441

[B3] Bae J. S. Koh I. Cheong H. S. Seo J. M. Kim D. Y. Oh J. T. (2016). A genome-wide association analysis of chromosomal aberrations and Hirschsprung disease. Transl. Res. 177, 31–40. 10.1016/j.trsl.2016.06.001 27370899

[B4] Britsch S. Li L. Kirchhoff S. Theuring F. Brinkmann V. Birchmeier C. (1998). The ErbB2 and ErbB3 receptors and their ligand, neuregulin-1, are essential for development of the sympathetic nervous system. Genes Dev. 12 (12), 1825–1836. 10.1101/gad.12.12.1825 9637684 PMC316903

[B5] Chatterjee S. Kapoor A. Akiyama J. A. Auer D. R. Lee D. Gabriel S. (2016). Enhancer variants synergistically drive dysfunction of a gene regulatory network in Hirschsprung disease. Cell 167 (2), 355–368. 10.1016/j.cell.2016.09.005 27693352 PMC5113733

[B6] Chatterjee S. Karasaki K. M. Fries L. E. Kapoor A. Chakravarti A. (2021). A multi-enhancer RET regulatory code is disrupted in Hirschsprung disease. Genome Res. 31 (12), 2199–2208. 10.1101/gr.275667.121 34782358 PMC8647834

[B7] Chen F. Yuan H. Wu W. Chen S. Yang Q. Wang J. (2019). Three additional de novo CTCF mutations in Chinese patients help to define an emerging neurodevelopmental disorder. Am. J. Med. Genet. C Semin. Med. Genet. 181 (2), 218–225. 10.1002/ajmg.c.31698 30893510

[B8] Ciarlo C. Kaufman C. K. Kinikoglu B. Michael J. Yang S. C D. A. (2017). A chemical screen in zebrafish embryonic cells establishes that Akt activation is required for neural crest development. Elife 6, e29145. 10.7554/eLife.29145 28832322 PMC5599238

[B9] Cummings C. T. Rowley M. J. (2022). Implications of dosage deficiencies in CTCF and cohesin on genome organization, gene expression, and human neurodevelopment. Genes (Basel) 13 (4), 583. 10.3390/genes13040583 35456389 PMC9030571

[B10] Emison E. S. McCallion A. S. Kashuk C. S. Bush R. T. Grice E. Lin S. (2005). A common sex-dependent mutation in a RET enhancer underlies Hirschsprung disease risk. Nature 434 (7035), 857–863. 10.1038/nature03467 15829955

[B11] Fadista J. Lund M. Skotte L. Geller F. Nandakumar P. Chatterjee S. (2018). Genome-wide association study of Hirschsprung disease detects a novel low-frequency variant at the RET locus. Eur. J. Hum. Genet. 26 (4), 561–569. 10.1038/s41431-017-0053-7 29379196 PMC5891499

[B12] Fleming M. S. Li J. J. Ramos D. Li T. Talmage D. A. Abe S. I. (2016). A RET-ER81-NRG1 signaling pathway drives the development of pacinian corpuscles. J. Neurosci. 36 (40), 10337–10355. 10.1523/jneurosci.2160-16.2016 27707970 PMC5050328

[B13] Garcia-Barcelo M. M. Tang C. S. Ngan E. S. Lui V. C. Chen Y. So M. T. (2009). Genome-wide association study identifies NRG1 as a susceptibility locus for Hirschsprung's disease. Proc. Natl. Acad. Sci. U. S. A. 106 (8), 2694–2699. 10.1073/pnas.0809630105 19196962 PMC2650328

[B14] Garratt A. N. Voiculescu O. Topilko P. Charnay P. Birchmeier C. (2000). A dual role of erbB2 in myelination and in expansion of the schwann cell precursor pool. J. Cell Biol. 148 (5), 1035–1046. 10.1083/jcb.148.5.1035 10704452 PMC2174554

[B15] Glenn T. D. Talbot W. S. (2013). Signals regulating myelination in peripheral nerves and the Schwann cell response to injury. Curr. Opin. Neurobiol. 23 (6), 1041–1048. 10.1016/j.conb.2013.06.010 23896313 PMC3830599

[B16] Gui H. Tang W. K. So M. T. Proitsi P. Sham P. C. Tam P. K. (2013). RET and NRG1 interplay in Hirschsprung disease. Hum. Genet. 132 (5), 591–600. 10.1007/s00439-013-1272-9 23400839

[B17] Gunadi, Kalim A. S. Iskandar K. Marcellus, Puspitarani D. A. Diposarosa R. (2023). Exome sequencing identifies novel genes and variants in patients with Hirschsprung disease. J. Pediatr. Surg. 58 (4), 723–728. 10.1016/j.jpedsurg.2022.11.011 36586783

[B18] Gunadi, Kalim A. S. Marcellus, Budi N. Y. P. Iskandar K. (2022). The impact of NRG1 expressions and methylation on multifactorial Hirschsprung disease. BMC Pediatr. 22 (1), 216. 10.1186/s12887-022-03287-1 35443634 PMC9019992

[B19] Gunadi, Iskandar K. Makhmudi A. Kapoor A. (2019). Combined genetic effects of RET and NRG1 susceptibility variants on multifactorial Hirschsprung disease in Indonesia. J. Surg. Res. 233, 96–99. 10.1016/j.jss.2018.07.067 30502294

[B20] Gunadi, Kapoor A. Ling A. Y. Rochadi, Makhmudi A. Herini E. S. Sosa M. X. (2014). Effects of RET and NRG1 polymorphisms in Indonesian patients with Hirschsprung disease. J. Pediatr. Surg. 49 (11), 1614–1618. 10.1016/j.jpedsurg.2014.04.011 25475805 PMC4258000

[B21] Guo Y. Zhang W. He R. Zheng C. Liu X. Ge M. (2022). Investigating the association between rs2439302 polymorphism and thyroid cancer: A systematic review and meta-analysis. Front. Surg. 9, 877206. 10.3389/fsurg.2022.877206 35558387 PMC9086625

[B22] He H. Li W. Liyanarachchi S. Wang Y. Yu L. Genutis L. K. (2018). The role of NRG1 in the predisposition to papillary thyroid carcinoma. J. Clin. Endocrinol. Metab. 103 (4), 1369–1379. 10.1210/jc.2017-01798 29121253 PMC6018707

[B23] Heermann S. Schwab M. H. (2013). Molecular control of schwann cell migration along peripheral axons: Keep moving. Cell Adh Migr. 7 (1), 18–22. 10.4161/cam.22123 23076214 PMC3544780

[B24] Hosseini-Jangjou S. H. Dastgheib S. A. Aflatoonian M. Amooee A. Bahrami R. Salehi E. (2021). Association of neuregulin 1 rs7835688 G > C, rs16879552 T > C and rs2439302 G > C polymorphisms with susceptibility to non-syndromic hirschsprung's disease. Fetal Pediatr. Pathol. 40 (3), 198–205. 10.1080/15513815.2019.1692113 31738640

[B25] Huang T. Hou Y. Wang X. Wang L. Yi C. Wang C. (2022). Direct interaction of Sox10 with cadherin-19 mediates early sacral neural crest cell migration: Implications for enteric nervous system development defects. Gastroenterology 162 (1), 179–192.e11. 10.1053/j.gastro.2021.08.029 34425092

[B26] Jiang Q. Arnold S. Heanue T. Kilambi K. P. Doan B. Kapoor A. (2015). Functional loss of semaphorin 3C and/or semaphorin 3D and their epistatic interaction with ret are critical to Hirschsprung disease liability. Am. J. Hum. Genet. 96 (4), 581–596. 10.1016/j.ajhg.2015.02.014 25839327 PMC4385176

[B27] Kapoor A. Jiang Q. Chatterjee S. Chakraborty P. Sosa M. X. Berrios C. (2015). Population variation in total genetic risk of Hirschsprung disease from common RET, SEMA3 and NRG1 susceptibility polymorphisms. Hum. Mol. Genet. 24 (10), 2997–3003. 10.1093/hmg/ddv051 25666438 PMC4406299

[B28] Kapoor A. Nandakumar P. Auer D. R. Sosa M. X. Ross H. Bollinger J. (2021). Multiple, independent, common variants at RET, SEMA3 and NRG1 gut enhancers specify Hirschsprung disease risk in European ancestry subjects. J. Pediatr. Surg. 56 (12), 2286–2294. 10.1016/j.jpedsurg.2021.04.010 34006365 PMC8526751

[B29] Karim A. Tang C. S. Tam P. K. (2021). The emerging genetic landscape of Hirschsprung disease and its potential clinical applications. Front. Pediatr. 9, 638093. 10.3389/fped.2021.638093 34422713 PMC8374333

[B30] Lai F. P. Lau S. T. Wong J. K. Gui H. Wang R. X. Zhou T. (2017). Correction of hirschsprung-associated mutations in human induced pluripotent stem cells via clustered regularly interspaced short palindromic repeats/cas9, restores neural crest cell function. Gastroenterology 153 (1), 139–153. 10.1053/j.gastro.2017.03.014 28342760

[B31] Leung A. K. C. Leung A. A. C. (2019). Evaluation and management of the child with hypothyroidism. World J. Pediatr. 15 (2), 124–134. 10.1007/s12519-019-00230-w 30734891

[B32] Luzón-Toro B. Torroglosa A. Núñez-Torres R. Enguix-Riego M. V. Fernández R. M. de Agustín J. C. (2012). Comprehensive analysis of NRG1 common and rare variants in Hirschsprung patients. PLoS One 7 (5), e36524. 10.1371/journal.pone.0036524 22574178 PMC3344894

[B33] Lyons D. A. Pogoda H. M. Voas M. G. Woods I. G. Diamond B. Nix R. (2005). erbb3 and erbb2 are essential for schwann cell migration and myelination in zebrafish. Curr. Biol. 15 (6), 513–524. 10.1016/j.cub.2005.02.030 15797019

[B34] Meyer D. Birchmeier C. (1995). Multiple essential functions of neuregulin in development. Nature 378 (6555), 386–390. 10.1038/378386a0 7477375

[B35] Morris J. K. Lin W. Hauser C. Marchuk Y. Getman D. Lee K. F. (1999). Rescue of the cardiac defect in ErbB2 mutant mice reveals essential roles of ErbB2 in peripheral nervous system development. Neuron 23 (2), 273–283. 10.1016/s0896-6273(00)80779-5 10399934

[B36] Ngan E. S. Garcia-Barceló M. M. Yip B. H. Poon H. C. Lau S. T. Kwok C. K. (2011). Hedgehog/Notch-induced premature gliogenesis represents a new disease mechanism for Hirschsprung disease in mice and humans. J. Clin. Invest. 121 (9), 3467–3478. 10.1172/jci43737 21841314 PMC3163945

[B37] Perlin J. R. Lush M. E. Stephens W. Z. Piotrowski T. Talbot W. S. (2011). Neuronal Neuregulin 1 type III directs Schwann cell migration. Development 138 (21), 4639–4648. 10.1242/dev.068072 21965611 PMC3190382

[B38] Pezzini F. Bettinetti L. Di Leva F. Bianchi M. Zoratti E. Carrozzo R. (2017). Transcriptomic profiling discloses molecular and cellular events related to neuronal differentiation in SH-SY5Y neuroblastoma cells. Cell Mol. Neurobiol. 37 (4), 665–682. 10.1007/s10571-016-0403-y 27422411 PMC11482124

[B39] Phusantisampan T. Sangkhathat S. Phongdara A. Chiengkriwate P. Patrapinyokul S. Mahasirimongkol S. (2012). Association of genetic polymorphisms in the RET-protooncogene and NRG1 with Hirschsprung disease in Thai patients. J. Hum. Genet. 57 (5), 286–293. 10.1038/jhg.2012.18 22377709

[B40] Pingault V. Zerad L. Bertani-Torres W. Bondurand N. (2022). SOX10: 20 years of phenotypic plurality and current understanding of its developmental function. J. Med. Genet. 59 (2), 105–114. 10.1136/jmedgenet-2021-108105 34667088 PMC8788258

[B41] Pu J. Tang S. Tong Q. Wang G. Jia H. Jia Q. (2017). Neuregulin 1 is involved in enteric nervous system development in zebrafish. J. Pediatr. Surg. 52 (7), 1182–1187. 10.1016/j.jpedsurg.2017.01.005 28190554

[B42] Rogounovitch T. I. Bychkov A. Takahashi M. Mitsutake N. Nakashima M. Nikitski A. V. (2015). The common genetic variant rs944289 on chromosome 14q13.3 associates with risk of both malignant and benign thyroid tumors in the Japanese population. Thyroid 25 (3), 333–340. 10.1089/thy.2014.0431 25562676

[B43] Salerno M. Improda N. Capalbo D. (2020). MANAGEMENT OF ENDOCRINE DISEASE Subclinical hypothyroidism in children. Eur. J. Endocrinol. 183 (2), R13–R28. 10.1530/eje-20-0051 32580145

[B44] Shea G. K. Tai E. W. Leung K. H. Mung A. K. Li M. T. Tsui A. Y. (2020). Juxtacrine signalling via Notch and ErbB receptors in the switch to fate commitment of bone marrow-derived Schwann cells. Eur. J. Neurosci. 52 (5), 3306–3321. 10.1111/ejn.14837 32460437

[B45] Sheng Q. Liu X. Fleming E. Yuan K. Piao H. Chen J. (2010). An activated ErbB3/NRG1 autocrine loop supports *in vivo* proliferation in ovarian cancer cells. Cancer Cell 17 (3), 298–310. 10.1016/j.ccr.2009.12.047 20227043 PMC2897158

[B46] Shi L. Bergson C. M. (2020). Neuregulin 1: An intriguing therapeutic target for neurodevelopmental disorders. Transl. Psychiatry 10 (1), 190. 10.1038/s41398-020-00868-5 32546684 PMC7297728

[B47] Tang C. S. Gui H. Kapoor A. Kim J. H. Luzón-Toro B. Pelet A. (2016). Trans-ethnic meta-analysis of genome-wide association studies for Hirschsprung disease. Hum. Mol. Genet. 25 (23), 5265–5275. 10.1093/hmg/ddw333 27702942 PMC6078638

[B48] Tang C. S. Karim A. Zhong Y. Chung P. H. Tam P. K. (2023). Genetics of Hirschsprung's disease. Pediatr. Surg. Int. 39 (1), 104. 10.1007/s00383-022-05358-x 36749416

[B49] Tang C. S. Li P. Lai F. P. Fu A. X. Lau S. T. So M. T. (2018). Identification of genes associated with Hirschsprung disease, based on whole-genome sequence analysis, and potential effects on enteric nervous system development. Gastroenterology 155 (6), 1908–1922. 10.1053/j.gastro.2018.09.012 30217742

[B50] Tang C. S. Tang W. K. So M. T. Miao X. P. Leung B. M. Yip B. H. (2011). Fine mapping of the NRG1 Hirschsprung's disease locus. PLoS One 6 (1), e16181. 10.1371/journal.pone.0016181 21283760 PMC3024406

[B51] Tang W. Li B. Xu X. Zhou Z. Wu W. Tang J. (2012). Aberrant high expression of NRG1 gene in Hirschsprung disease. J. Pediatr. Surg. 47 (9), 1694–1698. 10.1016/j.jpedsurg.2012.03.061 22974608

[B52] Tilghman J. M. Ling A. Y. Turner T. N. Sosa M. X. Krumm N. Chatterjee S. (2019). Molecular genetic anatomy and risk profile of hirschsprung's disease. N. Engl. J. Med. 380 (15), 1421–1432. 10.1056/NEJMoa1706594 30970187 PMC6596298

[B53] Van Ho A. T. Hayashi S. Bröhl D. Auradé F. Rattenbach R. Relaix F. (2011). Neural crest cell lineage restricts skeletal muscle progenitor cell differentiation through Neuregulin1-ErbB3 signaling. Dev. Cell 21 (2), 273–287. 10.1016/j.devcel.2011.06.019 21782525

[B54] Wang Y. L. Feng S. H. Guo S. C. Wei W. J. Li D. S. Wang Y. (2013). Confirmation of papillary thyroid cancer susceptibility loci identified by genome-wide association studies of chromosomes 14q13, 9q22, 2q35 and 8p12 in a Chinese population. J. Med. Genet. 50 (10), 689–695. 10.1136/jmedgenet-2013-101687 23847140

[B55] Watson L. A. Wang X. Elbert A. Kernohan K. D. Galjart N. Bérubé N. G. (2014). Dual effect of CTCF loss on neuroprogenitor differentiation and survival. J. Neurosci. 34 (8), 2860–2870. 10.1523/jneurosci.3769-13.2014 24553927 PMC6608523

[B56] Xie H. Chen D. Gu W. Li W. Wang X. Tang W. (2023). Thyroid function screening and follow-up of children with abdominal distension in nanjing, China: A cross-sectional study. BMJ Open 13 (1), e070416. 10.1136/bmjopen-2022-070416 PMC9884940 36697039

[B57] Yang D. Yang J. Li S. Jiang M. Cao G. Yang L. (2017). Effects of RET, NRG1 and NRG3 polymorphisms in a Chinese population with Hirschsprung disease. Sci. Rep. 7, 43222. 10.1038/srep43222 28256518 PMC5335705

[B58] Yang X. Ji C. Liu X. Zheng C. Zhang Y. Shen R. (2022). The significance of the neuregulin-1/ErbB signaling pathway and its effect on Sox10 expression in the development of terminally differentiated Schwann cells *in vitro* . Int. J. Neurosci. 132 (2), 171–180. 10.1080/00207454.2020.1806266 32757877

[B59] Zeltner N. Lafaille F. G. Fattahi F. Studer L. (2014). Feeder-free derivation of neural crest progenitor cells from human pluripotent stem cells. J. Vis. Exp. 87, 51609. 10.3791/51609 PMC4204793 24893703

[B60] Zhang L. Yang X. Yue Y. Ye J. Yao Y. Fu Y. (2015). Cyclic mechanical stress modulates neurotrophic and myelinating gene expression of Schwann cells. Cell Prolif. 48 (1), 59–66. 10.1111/cpr.12151 25418681 PMC6496414

[B61] Zhang T. Gygi S. P. Paulo J. A. (2021). Temporal proteomic profiling of SH-SY5Y differentiation with retinoic acid using FAIMS and real-time searching. J. Proteome Res. 20 (1), 704–714. 10.1021/acs.jproteome.0c00614 33054241 PMC8210949

[B62] Zheng Y. Sun Y. Liu Y. Zhang X. Li F. Li L. (2018). The miR-31-SOX10 axis regulates tumor growth and chemotherapy resistance of melanoma via PI3K/AKT pathway. Biochem. Biophys. Res. Commun. 503 (4), 2451–2458. 10.1016/j.bbrc.2018.06.175 29969627

