# Supplementary material for: The interplay of common genetic variants NRG1 rs2439302 and RET rs2435357 increases the risk of developing Hirschsprung’s disease
Source: Front Cell Dev Biol. 2023 Jul 7;11:1184799. doi: 10.3389/fcell.2023.1184799 (PMC10361661; doi:10.3389/fcell.2023.1184799)
Supplement: Supplementary file 1 [file DataSheet1.PDF]

Sanger sequencing primers
NRG1 2439302 F
NRG1 2439302 R
RET 2435357 F
RET 2435357 R

qRT-PCR primers
hRET-F
hRET-R
hNRG1-F
hNRG1-R
hSOX10-F
hSOX10-R
ERBB3-F
ERBB3-R
CTCF-F
CTCF-R
GAPDH-F
GAPDH-R
zRET-F
zRET-R
zNRG1-F
zNRG1-R

Whole Mount in situ Hybridization probe primers
NRG1 probe F
NRG1 probe R
RET probe F
RET probe R
SOX10 probe F
SOX10 probe R

CATAGGAGAGTTAGGTGGCAAAGC
CAAGAATGGCCTAACACAATGTG
ACAATGTAATCAGCTGGGGCA
GGCCTGTGGATGACCATGTAA

ACAGGGGATGCAGTATCTGG
CTGGCTCCTCTTCACGTAGG
CCCAGTTGTTGGTTTCAGGG
TTTCTCCAGTGGCAAAGCCT
TTCGGGTGGATTCAGGGTAG
GAGTTCCGAGTTCCAGGGTC

CTGCGGCTTTTGTCTGTTCTAA
CTCCTCCATTTTCCCCCTCTAC
GGTGAAGGTCGGAGTCAACG
CAAAGTTGTCATGGATGACC
CGTCCCACTTTCTCAGACA
CCGCAAGATCCAGATAGTCTC
TCCCGTCCTCCACTGTCAC
ACCCACAAATGACACGTCC

AAACCTTCGGAGCGTGACAG
GCTGGTGGCTGTGTTGATGATG
GCCAACCGCAACTCCAGCTACT
AAGCATCAGATTGTACATTTCAT
CCTGCACAACGCCGAGCTCA
AGGGCTGTGACTCTGACCTGT
